# Relationship between online pornography consumption and beliefs about sexual violence among young adults

**DOI:** 10.1192/j.eurpsy.2026.10861

**Published:** 2026-07-31

**Authors:** B. Conceição, B. R. Maia

**Affiliations:** 1 Universidade Católica Portuguesa, Braga, Portugal; 2 Faculty of Philosophy and Social Sciences; Centre for Philosophical and Humanistic Studies, Universidade Católica Portuguesa, Braga, Portugal

## Abstract

**Introduction:**

Scientific literature suggests that higher levels of online pornography consumption are associated with more permissive attitudes toward sexual violence, particularly when the content consumed includes aggressive or non-consensual themes.

**Objectives:**

This study explored psychosocial correlates of online pornography consumption and the relationship between online pornography consumption and beliefs about sexual violence.

**Methods:**

The sample consisted of 50 university students, evenly distributed by sex, with an average age of 22.48 years (*SD* = 2.75; range = 18-30). A Sociodemographic Questionnaire, a Questionnaire on Internet Usage Patterns and Pornographic Content, the Cyber Pornography Use Inventory-9, the Dickman Impulsivity Inventory, and the Scale of Beliefs about Sexual Violence were administered.

**Results:**

The results revealed that participants were exposed to online pornography at an average age of 12.71 years (*SD* = 2.21; range = 8-18) and began consuming this content at 14.87 years (*SD* = 2.93; range = 8-25). Men reported significantly higher weekly (Md= 33.74, n= 25) and monthly (*Md* = 33.98, *n* =25) versus females (*Md* = 17.26, *n* =25; *Md* = 17.02, *n* = 25; *U* = 106.50; *U* = 100.50, *p*>.000), *r*=60; *r*=.61, respectivaley) and between consumption of online pornography and a self-perception of addiction (males *Md* = 32.78, *n* = 25; females *Md* = 18.22, n = 25, *U*= 130.50, *r* = .50). The age of exposure to online pornography was correlated with compulsivity regarding pornography addiction (*rs* = -.30*, *p* = .04). It was also found that dysfunctional impulsivity was correlated with compulsivity (*rs* = .30*, *p* = .03). Finally, a relationship was identified between self-perception of addiction to online pornography and beliefs about sexual violence (*rs* = .28*, *p* = .05).

**Conclusions:**

Online consumption of pornography is related to negative psychological adjustment and to attitudes toward sexual violence. Preventive actions are needed, raising awareness about the negative effects of online pornography consumption.

Project funded UIBD/00683/2020, FCT.

**Disclosure of Interest:**

B. Conceição: None Declared, B. Maia Grant / Research support from: Grant / Research support from: FCT UIBD00683/2020

